# Student Characteristics, Institutional Factors, and Outcomes in Higher Education and Beyond: An Analysis of Standardized Test Scores and Other Factors at the Institutional Level with School Rankings and Salary

**DOI:** 10.3390/jintelligence10020022

**Published:** 2022-04-01

**Authors:** Jonathan Wai, Bich Tran

**Affiliations:** 1 Department of Education Reform, University of Arkansas, Fayetteville, AR 72701, USA; btt009@uark.edu; 2 Department of Psychology, University of Arkansas, Fayetteville, AR 72701, USA

**Keywords:** student characteristics, higher education, standardized test scores, rankings, general cognitive aptitude

## Abstract

When seeking to explain the eventual outcomes of a higher education experience, do the personal attributes and background factors students bring to college matter more than what the college is able to contribute to the development of the student through education or other institutional factors? Most education studies tend to simply ignore cognitive aptitudes and other student characteristics—in particular the long history of research on this topic—since the focus is on trying to assess the impact of education. Thus, the role of student characteristics has in many ways been underappreciated in even highly sophisticated quantitative education research. Conversely, educational and institutional factors are not as prominent in studies focused on cognitive aptitudes, as these fields focus first on reasoning capacity, and secondarily on other factors. We examine the variance in student outcomes due to student (e.g., cognitive aptitudes) versus institutional characteristics (e.g., teachers, schools). At the level of universities, two contemporary U.S. datasets are used to examine the proportion of variance accounted for in various university rankings and long-run salary by student cognitive characteristics and institutional factors. We find that depending upon the ways the variables are entered into regression models, the findings are somewhat different. We suggest some fruitful paths forward which might integrate the methods and findings showing that teachers and schools matter, along with the broader developmental bounds within which these effects take place.

## 1. Introduction

When seeking to explain the eventual outcomes of a higher education experience, do the personal attributes and background factors students bring to college matter more than what the college is able to contribute to the development of the students through education or other institutional factors? A long line of work within the fields that study cognitive reasoning and aptitudes would suggest that student background characteristics, especially cognitive aptitude, is important to outcomes not only within college but well beyond it (e.g., Brown et al. 2021; Deary et al. 2007; Schmidt and Hunter 2004), whereas other fields, such as the those that specifically study higher education, emphasize more the role that various factors attributable to the institution might make in the performance and eventual achievement of students (e.g., Light 2001; Stinebrickner and Stinebrickner 2007).

Ultimately, it is very hard to disentangle student and background factors from what a college adds, and, in part, all studies on education depend on the factors and methodological approach a researcher wishes to emphasize (e.g., Huntington-Klein 2020; Schlotter et al. 2011; Singer 2019). Most education studies tend to simply ignore cognitive aptitudes and other student characteristics—in particular the long history of research on this topic—since the focus is usually on trying to assess the impact of education, and so the role of student characteristics has in many ways been forgotten, or perhaps even ignored (Maranto and Wai 2020). Conversely, educational and institutional factors are not as prominent in studies focused on cognitive aptitudes or abilities, as these fields focus first on reasoning capacity (Hunt 2009), and perhaps secondarily on the contribution of other factors.

In this paper, we study student characteristics and institutional factors at the level of institutions to assess whether the findings align with the past studies on cognitive aptitudes and student characteristics, but also to examine the role of institutional or other factors. The aim of our paper is not to clearly adjudicate between the two different perspectives but to consider how our approach provides a way of thinking about this problem in different ways. We first provide a historical overview focused on the role of cognitive aptitudes, as this contribution adds to that line of work, and it also illustrates that this ongoing discussion surrounding what factors matter for education has a thread that can be traced back decades. We emphasize that our view on cognitive abilities and aptitudes is that they are developed and that education is both a product of cognitive aptitudes and can enhance cognitive aptitudes (Hair et al. 2015; Lohman 1993; Ritchie and Tucker-Drob 2018; Snow 1996).

## 2. Brief Historical Review Focused on the Role of Cognitive Aptitudes

The importance of cognitive aptitudes for life outcomes has been widely replicated across the decades in numerous longitudinal samples globally (e.g., Brown et al. 2021; Deary et al. 2007). Developed cognitive aptitudes are especially important for learning in schools (Detterman 2016; Snow 1996) and for educational outcomes (e.g., Brown et al. 2021; Deary et al. 2007). Though the typical approach to studying the basic science of cognitive aptitudes is not to consider its role in applied or historical contexts, sometimes it is within such contexts that a greater understanding of how and where basic science may or may not hold is obtained. In this paper, therefore, we examine the role of student cognitive aptitude in education by examining the pattern of correlations and variance explained by cognitive aptitude with educational and occupation-related outcomes, and, conversely, the variance explained by institutional factors. First, we provide a historical review of studies at both the individual and group level—illustrating the replication of findings across the very different disciplinary perspectives of cognitive aptitude research and education policy research—and then introduce our empirical study focused on the aggregate level of colleges and universities.

In a landmark U.S. educational report (Coleman et al. 1966), data were collected on multiple grades and over 4000 public schools on aptitude and achievement test scores of students, along with surveys of schools and students for a total sample of over 645,000. This report uncovered that about 10% to 20% of the variance in student achievement was due to schools, about 80% to 90% was due to student characteristics themselves, and teacher quality accounted for about 1% of the variance (Detterman 2016). This report initiated a national discussion, and much educational research since that time has investigated whether these findings are replicable. This study was on a representative U.S. sample at the time of K-12 students and schools. Do these findings hold for samples at different points in time, for individual and aggregate samples, for studies using different methods, and for studies in different countries? And do these findings hold not only in K-12 education, but also in higher education?

Over the following half century, reviews of the findings of what has come to be known as the Coleman report have largely confirmed them (e.g., Detterman 2016; Gamoran and Long 2007; Jencks et al. 1972). Jencks et al. (1972) replicated the finding that much of the variance in student achievement was due to students, and a 40-year follow up of the Coleman report, which included data on developing countries, Gamoran and Long (2007) found that in countries with a per capita income above $16,000 the findings were replicated.

### 2.1. Using Other Methods: Twin Studies and a Natural Experiment

Other than large sample randomized controlled trials (RCTs), studies of twins are able to account for endogenous factors such as genetics in the estimation of how much of the variance in student achievement is due to students versus teachers or schools in education research (Asbury and Wai 2020; Byrne et al. 2010; Hart et al. 2021). In a recent study examining classroom-level influences on literacy and numeracy among twin samples in the U.S. and Australia (Grasby et al. 2019), the classroom accounted for about 2% to 3% of the variance in achievement. These authors cautioned that although these averaged results may be a lower bound estimation, and that their design could not detect classroom influences at the level of the individual student, their estimate was at odds with much of the global public discourse focused primarily on the influence of teachers and the classroom.

An unusual opportunity for a natural experiment arose in World War II, due to the city of Warsaw, in Poland, being destroyed. The government assigned residents randomly in the newly reconstructed city. Firkowska et al. (1978) collected general cognitive aptitude data (Raven’s matrices) in addition to parent education and occupation for most of the students born in 1963 in Warsaw. When breaking down the variance in Raven’s scores due to district, school, and family characteristics, the authors found that the variance due to schools was about 2.1%. Thus, this estimate is right in line with the twin studies. Though this was an unusual natural experiment, it should be noted that at least for most rigorous large sample educational RCTs in the U.S. and U.K., these studies tend to find very small or uninformative effects that are typically much smaller than the literature that does not typically randomize (e.g., Lortie-Forgues and Inglis 2019; Sims et al. 2020).

### 2.2. Estimates of the Teacher’s Contribution to Student Achievement

Studies using K-12 student-level administrative data in the U.S. on a sample of about 23 million students in the states of Florida and North Carolina across the decade studied (Chingos et al. 2014; Whitehurst et al. 2013) were able to estimate the proportion of variance in student achievement on test scores due to teachers at about 4% to 6.7%, due to schools at about 1.7% to 3%, due to districts at about 1.1% to 1.7%, and due to superintendents at about 0.3%. This shows that—at least when ignoring the contribution of students (and related background factors) to student achievement—teachers appear most influential, followed by schools, districts, and superintendents.

### 2.3. Estimates of Teacher and School Effects Using Methods Focused on Forward Causal Inference

This tendency of education research to neglect the contribution of students to student achievement is probably due, in large part, to the focus of the education research community on what variables they think they can change in the educational environment of the student (e.g., Schlotter et al. 2011; Singer 2019). We should note that, up to this point in our brief review, the focus has been on studies at both the individual and group level that examine the proportion of variance accounted for by students, teachers, and schools. Additionally, we have summarized studies by treating ability and achievement tests as somewhat interchangeable, but there are debates around what is measured by large-scale international assessments such as PISA with regard to cognitive aptitudes versus learning outcomes (e.g., Baumert et al. 2009; Engelhardt et al. 2021; Rindermann 2007).

Gelman and Imbens (2013) explained that reverse causal questions are questions about the unknown causes of known effects, whereas forward causal inference requires estimating the unknown effects of known causes. Thus, in the literature reviewed so far, we are estimating the known variance proportions accounted for by student, teacher, and school sources without having a research design that can tell us what are the specific causes. However, forward causal questions would take the form of something like “What is the causal effect of having an effective teacher for one year on students’ academic outcomes?” (Wai and Bailey 2021), and to answer such a question policy researchers might use a random or quasi-random assignment of students to different teachers and assess the impact of this on outcomes. Much of the time outcomes are changes in test scores in the short run (for a review see Goldhaber 2015), but sometimes the effects of teachers can persist for years, such as on earnings (e.g., Chetty et al. 2011, 2014), and the differential effects of schooling environments can also influence short- and long-run outcomes (e.g., Atteberry and McEachin 2020; Chetty et al. 2014; Dynarski et al. 2013; Wolf 2019). 

Thus, the approach taken in this paper focused on reverse causal questions, providing some of the fuzzy boundaries around expectations of what teachers or schools might be able to contribute to the eventual achievement of students, but it does not necessarily take away from the utility of teachers and schools in improving student achievement and outcomes within reasonable bounds. The largest threat in most approaches from the economics of education is selection bias, which is even stated by education economists and policy researchers as cognitive abilities (e.g., Schlotter et al. 2011). For example, if students with higher developed cognitive aptitude are selected into a given program, it becomes unclear whether that higher aptitude, the program, or something else is causing later outcomes for those students. It makes sense for integrative understanding to use both or even additional approaches as complimentary tools to understanding the role of students, teachers, and schools in student achievement and what interventions may be cost-effective and beneficial relative to counterfactuals.

### 2.4. Estimates of the Students’ Contribution to Student Achievement

Up to this point we have focused on reviewing studies looking at the variance in student achievement accounted for by schools or teachers, but Coleman et al. (1966) estimated that roughly 80% to 90% of student achievement variance was due to students and related background factors (Detterman 2016). What about studies that estimate the student’s contribution to student achievement? Deary et al. (2007) examined 13,248 English school children who were tested on The Cognitive Abilities Test at age 11 and took General Certificate of Secondary Education (GCSE) tests around age 15 or 16. The correlation between the academic achievement general factor and the cognitive aptitude general factor was 0.81. Kaufman et al. (2012) examined 2520 participants who took the Kaufman intelligence and achievement tests and 4969 participants who took the Woodcock–Johnson intelligence and achievement tests. The overall average correlation between the academic achievement general factor and the cognitive aptitude general factor was 0.83. Thus, in both these studies in the U.K. and U.S., respectively, general cognitive aptitude accounted for roughly two-thirds of the variance in academic achievement.

### 2.5. Higher Education

So far, we have reviewed findings in K-12 education. But what about higher education? At the individual level, Angoff and Johnson (1990) used a sample of 7954 students from 292 institutions who had taken the SAT and then about a half-decade later had taken the GRE. They used SAT math, college major, and gender and were able to predict 93% of the variance in GRE math scores. This means that roughly 7% of the variance in student achievement could be attributable to the institution the student attended. Additionally, Dale and Krueger (2002) examined the role of the selectivity of the institution in impacting long-run earnings using large samples and controlling for multiple confounders. Overall, once the SAT of the school was accounted for, there was no connection between the selectivity of the institution attended and long-run earnings overall. Taken along with arguments from other scholars that the value of higher education may not be so much about the institution one attends (e.g., Caplan 2018; Wolf 2003), this provides similar findings at the level of higher education as reviewed for K-12 education.

*Value-added in higher education*. As more attention has been drawn to accountability and transparency in higher education in the past decade, many researchers turn to using the value-added methodology to determine the aspects that higher education may add to economic opportunities (Roohr et al. 2021; Kulkarni and Rothwell 2015). However, there are certain challenges in measuring value-added in higher education, particularly using administrative data (Cunha and Miller 2014). Such challenges include the lack of year-on-year standardized tests, the lack of longitudinal student-level outcomes, concerns of self-selection into college and university, and the mismatch between students’ specialization and outcome measures. Cunha and Miller (2014) proposed a simple model to estimate the value-added of individual institutions that include pre-enrollment characteristics, unobserved differences in student’s profile and preferences captured by applications and acceptances, and fixed effects for the college they enrolled in. In our current study, unfortunately, we do not have access to student-level characteristics. Instead, we focus on institutional-level characteristics.

## 3. This Study

For this study, we link the higher education literature with the cognitive aptitude literature by examining the proportion of variance accounted for by students versus institutional characteristics at the level of colleges and universities in the U.S., at least to the extent that standardized test scores such as the SAT or ACT can be used to tap such student characteristics. Before describing our specific research design and questions in more detail, we explain our perspective on the measurement of student cognitive characteristics that helps unify and integrate the findings that have come from various disciplinary perspectives. The key is the measurement of student cognitive characteristics, in particular the measurement of general cognitive aptitude.

### 3.1. Measurement of Student Cognitive Characteristics

The measurement of student cognitive characteristics, in particular through tests or assessments aimed at measuring cognitive aptitudes and their use in the selection of various kinds, has a long history (Binet and Simon 1905; Spearman 1904; for reviews, see Detterman 2016; Thorndike and Lohman 1990). Even as early as 200 B.C., for example, the Chinese arguably selected for cognitive aptitude through the use of Civil Service Examinations, and even today, the *gaokao*, or national college entrance examination in China, is viewed as a measure of student cognitive aptitude (Li et al. 2012). Though there are multiple cognitive aptitudes, a general working consensus around the hierarchical model of cognitive aptitudes has emerged that recognizes general cognitive aptitude at the apex along with more narrow aptitudes below that (Carroll 1993).

There is also extensive research on the overlap between aptitude and achievement tests, and, in fact, Kelley (1927; c.f. Coleman and Cureton 1954, p. 347) introduced the idea of the jangle fallacy as “the use of two separate words or expressions covering, in fact, the same basic situation, but sounding different, as though they were in truth different,” referring to the significant measurement overlap between group cognitive aptitude tests and school achievement tests. Indeed, research has shown that cognitive g and academic achievement g are roughly the same from a measurement standpoint (Deary et al. 2007; Kaufman et al. 2012), that g is measured by nearly any challenging cognitive test with a diversity of tests and item types (e.g., Chabris 2007; Ree and Earles 1991), and that even when test designers intended to measure other aptitudes and achievements, g is uncovered (e.g., Johnson et al. 2004; Schult and Sparfeldt 2016; Steyvers and Schafer 2020). Given this broadly replicated finding, it should come as no surprise to those who acknowledge the body of research on cognitive aptitudes that both the SAT and ACT have largely been found to be measures of g (e.g., Frey and Detterman 2004; Koenig et al. 2008). We should make clear that we are discussing here a very specific, yet central, dimension of student characteristics, that such characteristics can encompass cognitive, noncognitive, and other attributes associated with the student (e.g., Wai and Lakin 2020), and that we view these attributes as developed. As Detterman (2016) puts it, student characteristics can be broadly characterized as things that go with the student when they leave a school, which include aspects associated with income and parental education level (Hair et al. 2015).

### 3.2. Analytic Plan

We build upon this body of work that spans decades and different disciplinary approaches by examining, at the college or university level in the U.S., the proportion of variance accounted for in various college rankings and early to mid-career salary by student characteristics as indicated by SAT or ACT scores, as well as various institutional factors. We draw from two longitudinal databases at two different points in time which measured these factors somewhat differently. The first database was drawn largely from the U.S. News & World Report, along with salary data collected by PayScale. Both sources date from 2014. The second database was drawn from College Scorecard data in 2017–2018 (U.S. Department of Education College Scorecard 2017–2018). Broadly, we seek to examine what proportion of variance student characteristics (as indicated by general cognitive aptitude) account for in typical college and university outcomes, such as rankings and salary, and also to estimate, after cognitive aptitude is taken into account, what proportion of variance in rankings and salary remain for institutional factors to account for among the explainable variance. We also take the flipside perspective and examine the role of what cognitive aptitude adds after accounting for a wide range of institutional factors. We use these two datasets along with Lykken’s (1968) approach of constructive replication—the idea of preserving focal constructs in each database but varying construct-irrelevant aspects—to investigate whether findings replicate across the two datasets, and also across the decades of literature reviewed at multiple levels of education.

## 4. Method

### 4.1. Data and Analytic Sample

We use two datasets for this study at different time points and measurement of different outcomes to attempt to see if the findings replicate. The first dataset was compiled in 2014 from the U.S. News website using a premium account for full access as well as public data from PayScale. The second dataset was drawn from the College Scorecard database from 2017–2018. This dataset is free and available to access and download via https://collegescorecard.ed.gov/data/ (accessed on 23 March 2022). Table 1 shows each of the comparable variables used in this study, which were purposefully selected to represent student (i.e., SAT or ACT scores) and various institutional factors, of which we discuss how we selected for inclusion in the next section. After matching all observations by university names, we had a total of 1271 universities and colleges in the College Scorecard dataset in 2017–2018, and 883 universities and colleges in the U.S. News dataset.

### 4.2. Variables

*Student characteristics*. We used average SAT and ACT scores at the institutional level as a proxy for students’ average general cognitive aptitude level (e.g., Frey and Detterman 2004; Koenig et al. 2008; see Table 1 for a description of variables). As in prior work (e.g., Wai et al. 2018), for the U.S. News reported scores this average was computed by translating ACT scores to SAT scores using a conversion table and then taking an average of the 25th and 75th percentile scores (what universities report to U.S. News) to create an SAT average for all schools with data. For the College Scorecard database, an SAT average which was already computed was used.

*Outcomes*. We used average income/salary at early and mid-career points at the institutional level as a proxy for short-term and long-term outcomes of students, as well as university rankings on various measures (see Table 1). College and university rankings are conducted by numerous publications seeking to quantify differences in quality between schools in diverse ways. We drew from rankings data in prior work (Wai et al. 2018) looking at U.S. News national university and U.S. News liberal arts college rankings, a critical thinking ranking (using a measure of critical thinking known as the CLA+), a Lumosity brain games ranking which included data from different colleges and universities whose students had played their brain training games, Times Higher Education (THE) world and U.S. rankings, and a revealed preference ranking (Avery et al. 2013, p. 425), which ranked schools based on “the colleges students prefer when they can choose among them”. Income/salary is a clear and objective occupational outcome metric which is often used in evaluating the role of higher education but has also been linked to cognitive aptitudes (Brown et al. 2021; Judge et al. 2010; Schmidt and Hunter 2004). In this study, we could only use the THE U.S. ranking and the Lumosity brain games ranking in our analysis with sufficient sample size (N~200), where both institutional factors and student cognitive characteristics were examined. 

*Institutional characteristics*. Our institutional-level variables included data on tuition and fees, admission rate, university resources, cost of attending (including room and board), and diversity (see Table 1). The role of tuition and fees and the overall cost of attending university may matter for students in terms of the time they spend studying versus the time they must work in addition to studying. For example, some studies suggest students that attend colleges with higher tuition are more likely to work while studying (Neill 2015). However, whereas Light (2001) suggested that working while studying yielded higher future wages, Stinebrickner and Stinebrickner (2007) noted that additional study time was associated with higher academic performance. Given that work-study and additional study time may come into conflict with one another, it is unclear how tuition and the cost of attending may affect student outcomes with significant confounding factors coming from students’ choice of tracks to complete their degrees (Neyt et al. 2018), in addition to student cognitive aptitudes, which predict numerous long-term outcomes throughout life (e.g., Brown et al. 2021; Deary et al. 2007; Schmidt and Hunter 2004). 

School facilities and intellectual resources as well as quality are proxied by endowment, number of faculty, faculty–student ratio, enrollment, and admission rate, though it is unclear whether these resources are crucial for student achievement after graduation (Caplan 2018; Dale and Krueger 2002; Wolf 2003). Some studies suggest that educational expenditure and university resources are modestly related to student learning outcomes for certain groups of students, for example freshmen (Pike et al. 2011; Winitzky-Stephens and Pickavance 2017). Instructor quality might also contribute to student outcomes. Cash et al. (2017) studied the relationship between perceptions and resources of large universities using a multidimensional approach to survey students and instructors, and found that instructors were the key determinant for students’ outcomes. In particular, in large universities, to make a class feel small to promote student achievement, the researchers argued effort should be placed on instructor quality and course structure as determined by instructors (Cash et al. 2017). Other university resources, including access to library and electronic databases—which correlate with university financial resources—also have been found to have a positive correlation with student performance (Montenegro et al. 2016).

Researchers have also studied the relationship between classroom diversity as well as diversity courses and students’ cognitive outcomes (Roksa et al. 2017; Gottfredson et al. 2008; Bowman 2013). Roksa et al. (2017), leveraging a longitudinal study following three cohorts of students from their first to their last year in college, found that diversity experiences were correlated with student cognitive outcomes, with the correlation being stronger for white students compared with non-white students. Gottfredson et al. (2008) studied 6800 incoming law students in a nationally representative sample and found that classroom diversity had a moderate positive effect on students “openness and enthusiasm to learn new ideas and perspectives” (p. 85). Bowman (2013) studied a longitudinal sample of 8615 first-year undergraduates at 49 universities and found that frequent diversity interactions were associated with gains in students’ outcomes including leadership skills, psychological well-being, intellectual engagement, and intercultural effectiveness. However, Martins and Walker (2006) found that students’ unobservable characteristics moderated student achievement significantly even when controlling for attendance, class size, peers, and teachers. With the interest in diversity demonstrated in the literature, in this study we used a college diversity index as a proxy for college diversity. This index, on a scale from 0 to 100, was obtained from the Chronicle of Higher Education database (The Chronicle of Higher Education forthcoming) through a membership subscription (https://www.chronicle.com/package/diversity/ accessed on 23 March 2022). 

In the College Scorecard data, we had more than 6000 observations. However, there are also significant missing observations in this dataset. For example, among more than 6000 institutions, only 1300 of them reported average SAT scores at the institutional level. Some patterns we observed in this dataset are: (1) the average SAT score is 1060, (2) there is a wide range of admission rates, total enrollment, faculty salary, and cost to attend, for example; (3) the majority of institutions are private-for-profit. For the U.S. News dataset, we faced a less significant issue of missing data. We see that the majority of institutions in this dataset are private-not-for-profit institutions. More details can be found in Appendix A Table A1 and Table A2.

### 4.3. Statistical Methods

We used ordinary least squared (OLS) techniques to analyze the relationship between student aptitude, institutional factors, and student outcomes. First, we ran a model with only SAT scores on student outcomes to uncover the variance explained by student characteristics or cognitive aptitude alone (The Table A1, Table A2 and Table A3 in Appendix A include the full set of outcomes and results based on the broader sample of colleges and universities not restricted based on institutional factor availability for all cases). Second, we only used institutional variables which accounted for the cost of attending, university types (private, public, and for-profit), locale (urban, suburban, rural, and city), and regions (seven designated regions) in the model to obtain the percent variance explained by institutional characteristics. Third, we included both SAT and institutional factors in our final model. We added controls for university types, locales, and regions to account for plausible differences between types of universities in terms of their internal policies, as well as regional and locale differences that may contribute to variations in institutional outcomes. Our models are as follows:

Model 1: $outcome_{i}=\beta_{0}+\beta_{1}SAT_{i}+\varepsilon_{i}$, where $outcome_{i}$ is the respective outcome for school i and $SAT_{i}$ is the average SAT score for that school.

Model 2: $outcome_{i}=\beta_{0}+\beta_{1}I_{i}+\Omega_{i}+\pi_{i}+\varepsilon_{i}$, where $I_{i}$ is a matrix of institutional-level variables, as mentioned, Ω is type of university fixed-effects, and Ω is location fixed effects.

Model 3: $outcome_{i}=\beta_{0}+\beta_{1}SAT_{i}+\beta_{2}I_{i}+\Omega_{i}+\pi_{i}+\varepsilon_{i}$, is the combined model from (1) and (2) where we study the joint explained variance by including both the SAT score and institutional variables. Errors are clustered at the state level. 

Finally, to study the question of what explains the institutional level outcomes, including the ranking and average salary of graduates at early and mid-career points, we calculated two ratios. The ratio of the two robust R-squared values $\frac{R_{model\;1}^{2}}{R_{model\;3}^{2}}$ indicates how much of the respective outcome variance is explained by SAT scores when also accounting for institutional characteristics and average SAT scores. The ratio of the two robust R-squared values $\frac{R_{model\;2}^{2}}{R_{model\;3}^{2}}$ indicates how much of the respective outcome variance is explained by institutional factors when also accounting for SAT average score and institutional characteristics. We made sure that we used a sufficient sample size (N~200) for three models that included SAT average scores and institutional characteristic variables. We also dropped certain outliers in each faculty’s average monthly salary in the College Scorecard data. We dropped outliers by examining the variable’s distribution and summary statistics. We dropped observations that were beyond the lower and upper bounds (median +/− 1.5*inter-quartile range). Finally, we dropped data points with zero values in retention rates and admission rates in the two datasets.

In addition, we also included dominance analysis (DA) to determine the importance of independent variables (for further details, see Grömping 2007; Luchman 2015, 2021). This additional analysis is to provide a picture of what factors contribute the most to our model fit statistic. particularly, DA provides a “theory-grounded method for ascribing components of a fit metric to multiple, correlated independent variables” (Luchman 2015, p. 10). 

## 5. Results

Table 2 and Table 3 present coefficients, standard errors, and R-squared values from OLS regressions. In model 1 and model 3 in Table 2, where the SAT average score at the institutional level was included, the estimated coefficients were statistically significant, indicating that the SAT average score was a statistically significant predictor of students’ short-term and long-term outcomes measured by salary. This result was replicated across the two datasets. Similarly, when looking at institutional rankings as reported in Table 3, SAT average scores were also a statistically significant predictor of college ranking. The higher the average SAT score, the higher the institution’s score in both the THE U.S. ranking and the Lumosity ranking (rankings are reversed in order). 

Table 4 summarizes the proportion of variance explained in each respective outcome accounted for by average SAT scores even when accounting for institutional characteristics. Panel A presents data collected from the College Scorecard; Panel B represents data collected from U.S. News. In Panel A, we observe that by using the average SAT score only, we were able to account for 42% of the variation in the average salary six years out and 47% of the variation in the average salary ten years out. The explained variation in salary was smaller in Panel B. By using average SAT scores, we were able to explain 30% of the variance in the early-career salary and 41% of the variance in the mid-career salary. 

In both datasets, average SAT scores accounted for more variance in the institutions’ rankings than students’ outcomes. In Panel A, 53% of the variance in the institutions’ THE U.S. ranking and 51% of the variance in the Lumosity ranking were accounted for by the change in average SAT scores. Similarly, in Panel B, 56% of the variance in the THE U.S. ranking and 56% of the variance in the Lumosity ranking in the second dataset can be accounted for by the change in average SAT scores.

In Table 4 Model 2 *R*^2^, we only included selected institutional factors as predictors of students’ outcomes and institutional rankings. When comparing the value of *R*^2^ in Model 1 and Model 2, except for the explained variation in the Lumosity rankings using the U.S News dataset, institutional factors accounted for more variation in student outcomes and the THE U.S. ranking than the average SAT scores. In particular, looking at *R*^2^ values for Model 2, in the College Scorecard dataset, institutional factors explained 57% of the short-term salary (six years out) and 64% of the long-term salary (ten years out). In the 2014 U.S. News data, institutional factors accounted for 38% of the variance in the average early-career salary and 53% of the variance in the average mid-career salary at the institutional level. In terms of rankings, institutional factors could account for between 75% (in the College Scorecard data) and 75% (in the U.S. News data) of variance in the THE U.S. ranking, and between 59% (in the College Scorecard data) and 52% (in the U.S. News data) of the variance in the Lumosity ranking. 

When including both average SAT scores and institutional factors in the model, we observed increases in the explained variance of our outcome measures. We also examined the proportion of variance explained by calculating R-squared ratios. We compared two ratios: $\frac{R_{model\;1}^{2}}{R_{model3}^{2}}$ and $\frac{R_{model2\;}^{2}}{R_{model3}^{2}}$. We found that, across the two datasets, institutional factors (taken collectively) appear to explain a greater amount of variation in students’ average salary at early- and mid-career points and the institutions’ THE U.S. ranking. However, it is worth noting that by using the average SAT alone, we could already explain a large portion of the variation in students’ outcomes and institutions’ rankings compared to other institutional factors.

Finally, we report findings using the College Scorecard dataset in Table 5 and U.S. News data in Table 6. In our multiple regression model predicting salary outcomes using the College Scorecard data, the top three predictors for six-year salary were: % of students who received a Pell grant, average SAT score, and retention rate (see Table 5 Panel A). For ten-year salary, the top predictors were retention rate, average SAT score, and % of students receiving a Pell grant (see Table 5 Panel B). In the U.S. News data, the top three predictors for early-career salary were average SAT scores, average freshmen retention rate, and endowment (see Table 6 Panel A). For mid-career salary, the top predictors were average SAT score, average freshmen retention rate, and room and board cost (see Table 6 Panel B). 

Looking at rankings, specifically the THE U.S. ranking and Lumosity ranking, we found that the top three predictors of THE U.S. ranking using the College Scorecard data were completion rate, retention rate, and faculty salary (see Table 5 Panel C). For the Lumosity ranking the top three predictors were average SAT score, % of students who received a Pell grant, and retention rate (see Table 5 Panel D). Using the U.S. News data (see Table 6), we found the top three predictors for the THE U.S. ranking were retention rate, average SAT score, and total enrollment (see Panel C); the top predictors for the Lumosity ranking were average SAT score, retention rate, and endowment (see Panel D). Average SAT score and retention rate were the most significant predictors of both student long-term outcomes and institutional rankings across the two datasets.

## 6. Discussion

Overall, our findings aligned historically with much of the research on cognitive aptitudes and variance explained in outcomes, even after accounting for various institutional factors. However, this was from the perspective of cognitive aptitudes being the core variable of importance to consider as a starting point. On the flipside, when entering the multitude of institutional factors first into the regression model, these numerous variables collectively accounted for the majority of the variance in outcomes (in most cases larger than the proportion of variance accounted for by test scores alone), suggesting that institutional factors very likely do matter, in addition to student characteristics and cognitive aspects. Of course, test scores such as the SAT are just one short measure that students take prior to high school, so the fact that much of the variance in outcomes is captured by this singular measure should not be underemphasized. At the same time, this analysis illustrates that other institutional factors can matter collectively, and/or the contribution of student characteristics might be obscured or highlighted depending upon which variables one prioritizes in the research design and analysis. Depending upon the ways variables are prioritized and entered into regression models, findings can be quite different. 

In the remaining part of this discussion, though we fully acknowledge that institutional factors play an important role in addition to student characteristics, we discuss our findings that link to the historical focus of the academic field focused on cognitive aptitudes, and consider our findings in that broader context, and through the lens of cognitive aptitudes’ usefulness.

### 6.1. Limitations

A core limitation of this study is that our research design is in the form to address a reverse causal question where we cannot isolate causes. Thus, we likely have omitted variable biases. However, because one purpose of the study was to determine whether the proportions of variance in student achievement due to students or to institutional factors aligned with the large historical literature going back to Coleman et al. (1966; see Detterman 2016, for a review) at the level of colleges and universities, our approach is appropriate to test whether these findings could be replicated in contemporary U.S. samples. Another possible limitation is that our findings are at the group rather than individual level, and could potentially reflect the ecological fallacy (e.g., Piantadosi et al. 1988), however, Angoff and Johnson (1990) found similar findings as ours at the individual level. Another limitation is that the outcomes we examined were restricted to various school rankings and to salary, which are only a limited set of educational and occupational outcomes. University rankings are an imperfect outcome given that the decision to apportion weights to various aspects is quite variable and reflect the policy decision of the ranker. However, our findings were replicated across many different types of rankings, which reflect numerous weighting formulas (especially see Appendix A Table A1 through Table A3). Additionally, salary is often a core outcome used in evaluating colleges and universities (e.g., Dale and Krueger 2002), and thus the outcomes used are appropriate, but are limited to what we were able to access based on the datasets used. Relatedly, we also have the issue of missing data. Our data were collected from multiple sources that may not adequately synchronize with one another. Therefore, even though at some point we had more than 6000 observations, after running multiple regressions, we were down to 200–300 observations. Our findings, therefore, are not necessarily representative of the broader domain of institutions.

### 6.2. Findings Replicate and Extend Those in K-12 to Higher Education and Also Historically

Despite these important limitations, our findings illustrate contemporary replications across two U.S. datasets at different time points at the level of colleges and universities, with the many studies reviewed in K-12 education, and also historically. Overall, the proportion of variance accounted for by student characteristics as indicated by average SAT/ACT scores or general cognitive aptitude—even after accounting for various institutional factors—was quite consistent across not only typical college rankings but also a critical thinking ranking and a Lumosity brain games ranking (see Table A1, Table A2 and Table A3 in Appendix A for the full range of analyses of rankings, excluding institutional factor controls). This suggests that even measures intended to assess supposedly unique constructs such as critical thinking (e.g., Butler et al. 2017) may in fact end up largely overlapping with general reasoning. Additionally, brain games such as those from Lumosity, which were intended to improve cognitive aptitudes, may end up largely measuring a latent learning g factor (e.g., Steyvers and Schafer 2020), which aligns with other research showing that even video games may be measuring cognitive aptitudes (e.g., Quiroga et al. 2015, 2019). Given that various rankings, such as U.S. News, only lightly weight SAT/ACT scores in their ranking formula and yet such scores account for the majority of the variance in those rankings suggests that much of university quality may actually be due to student quality at the point of selection (e.g., Dale and Krueger 2002; Wai et al. 2018). Of course, this does not rule out various dimensions of university education or impact, such as brand of degree in helping improve employment prospects, among other factors, but does provide bounds around thinking of the contribution of *developed cognitive aptitudes at the point of testing* and institutional or other factors and their contributions to long-run outcomes.

The proportion of variance accounted for by SAT/ACT scores or general cognitive aptitude on long-run salary was replicated across the U.S. News and College Scorecard datasets which used two different measurements of salary. Overall, College Scorecard data showed that approximately 47% of the variance in salary a decade after graduation was accounted for by such test scores and U.S. News, and PayScale data showed that approximately 41% of the variance in salary at mid-career was accounted for by test scores. Findings for salary, even after accounting for institutional factors, were consistently replicated across different career time points and datasets, ranging from 72% up through 74%.

### 6.3. Part of Student Outcomes May Be Due to Selection, but Teachers and Institutions Still Matter

In a classic paper, Dale and Krueger (2002) showed that once SAT scores were accounted for, there were no differences in long-run salary for students attending a highly selective school compared to those who attended a less selective school. This indicated the importance of selection on student characteristics—especially cognitive aptitudes (see also Angoff and Johnson 1990). Overall, the findings from this study align with the Dale and Krueger (2002) findings suggesting the importance of cognitive aptitudes before college in predicting outcomes well after college (e.g., Lubinski and Benbow 2020). This also aligns with other literature on selective high schools showing that student selection effects, perhaps more than school quality, may be driving differences in outcomes (e.g., Abdulkadiroglu et al. 2014; Dobbie and Fryer 2014; Dynarski 2018), as well as scholars who have argued that much of the impact of college or university may be attributable to selection (Caplan 2018; Wolf 2003). It appears that cognitive aptitudes remain an important threat to selection bias in forward causal inference approaches, and a more careful consideration of how cognitive aptitudes are important across the lifespan in relation to educational interventions and other policies is in order.

Teachers and other institutional factors do matter (as we illustrated by entering institutional factors first rather than cognitive aptitudes in our models). However, at least from the broad empirical historical perspective of cognitive aptitudes research, how and the extent to which institutional and educational factors can matter is bounded in some ways by this broader pattern of student characteristics, accounting for a large portion of the variance in long-run student outcomes. For example, Chetty et al. (2011, 2014) illustrated that teacher effects can have causal impacts on long-run earnings, and rigorous work on the differential effects of the types of schooling environments shows that institutional effects matter (e.g., Atteberry and McEachin 2020; Chetty et al. 2014; Dynarski et al. 2013; Wolf 2019), which also aligns with our finding when entering institutional factors prior to cognitive aptitude tests. Additionally, a great deal of literature supports the idea that parents’ education level, earnings, and social capital are important to the development of eventual student success (e.g., for a summary see Egalite 2016; Hair et al. 2015; Heckman 2000). The wide range of variables we examined in this study may be picking up some of these factors, by proxy. And even though the diversity index, as part of the institutional factors control in this study, did not appear to be a major factor in student outcomes, there may be other values to diversity that are not necessarily quantifiable or achievement-outcome-related, such as simply being exposed to a wide range of people from a unique range of backgrounds and circumstances. More broadly, the resources that an institution holds—such as access to top professors, other highly talented students, opportunities for research, prestige of brand, or alumni networks—can vary widely alongside student cognitive quality, which may serve to further amplify the outcomes of graduates. This may be in part why in the U.S. roughly half of numerous leaders in society have graduated from just a handful of elite institutions and likely, by proxy, have high developed cognitive aptitudes (e.g., Wai 2013; Wai et al. 2018; Wai and Perina 2018).

### 6.4. Conclusions and Future Directions

Taken from the lens of cognitive aptitudes as being important, this paper replicated and extended findings in two contemporary U.S. datasets at the level of universities extending decades of research at many levels of education, suggesting that a large portion of the variance in student outcomes may be due to student characteristics—in particular developed cognitive aptitude. When coupled with the large literature showing that general reasoning is related to numerous outcomes across the lifespan (Brown et al. 2021; Deary et al. 2007; Kuncel et al. 2004; Schmidt and Hunter 2004), these findings suggest that across at least the last half century the contribution of students to long-run student achievement has been underappreciated in U.S. education (Detterman 2016; Maranto and Wai 2020), an omitted set of variables in education (Schmidt 2017). This may also highlight the neglect of U.S. education research and policymakers regarding general cognitive aptitudes and individual differences in students across a more comprehensive range of well-studied individual differences characteristics (Lubinski 2020; Revelle et al. 2011). Various cognitive and noncognitive aptitudes might be fruitfully developed by education, but should also be accounted for when helping students receive a differentiated education in schools throughout their developmental trajectory (e.g., Lakin and Wai 2022).

Some fruitful avenues to explore taking individual differences in aptitude into account for more optimal talent or human capital development might be to more carefully examine what aspects of education could improve intelligence (Ceci 1991; Snow 1996; Ritchie and Tucker-Drob 2018), which educational-intervention effects persist and fade out when accounting for intelligence (e.g., Bailey et al. 2020), and differentiating instruction to more closely match individual differences and characteristics of students (e.g., Lakin and Wai 2020). More broadly, this research highlights the need for the approach of asking reverse causal questions to be integrated with the approach focused on forward causal inference (Wai and Bailey 2021), for education economists and policy researchers to pay more attention to the established structure of cognitive aptitudes as a threat to selection bias using forward causal inference tools (Schlotter et al. 2011), in addition to appreciating a broader methodological approach and integration of research evidence which is often found across disciplinary boundaries (e.g., Singer 2019). Ultimately, whether one thinks student characteristics or institutional characteristics matter more is highly dependent upon what research lens and historical evidence one brings to the table in one’s sample, research design, and analytical approach.

## Figures and Tables

**Table 1:** Variable selection and description.

Dataset	U.S. News 2014	College Scorecard 2017–2018
Variables		
*Student characteristics*		
SAT score	Average SAT score	Average SAT score
Outcomes		
Earning	PayScale early-career	6 years after graduation
	PayScale mid-career	10 years after graduation
Ranking		
	*THE* U.S. Ranking	*THE* U.S. Ranking
	Lumosity Ranking	Lumosity Ranking
*Institutional factors*		
Cost of attending	Room and board	Cost per academic year
Admission, enrollment, and completion	Total enrollment	Total enrollment
	Acceptance rate	Admission rate
		Six-year completion rate
	Average freshmen retention rate	Retention at 4-year-institution
University resources	Endowment	Faculty average salary/month
	% Graduating students ever borrowed loan	% Students ever borrowed
	Average student debt	% Students received Pell grants
Diversity	Diversity index	Diversity index

**Table 2:** OLS regression coefficients of SAT/ACT and institutional factors predicting salary.

**Panel A: College Scorecard Data 2016–2017**						
	6-year Salary			10-year Salary		
	Model 1 (SAT)	Model 2 (Institutional factors)	Model 3 (SAT + institutional factors)	Model 1 (SAT)	Model 2 (Institutional factors)	Model 3 (SAT + institutional factors)
**Coefficient for SAT score**	44.228 ***	n/a	16.387 *	65.634 ***	n/a	22.039 *
	(3.767)		(7.955)	(5.299)		(9.651)
**N**	341	341	341	341	341	341
***R* ^2^**	0.419	0.569	0.587	0.470	0.641	0.653
**Panel B: *U.S. News* Ranking Data 2014**						
	Early-career Salary			Mid-career Salary		
	Model 1 (SAT)	Model 2 (Institutional factors)	Model 3 (SAT + institutional factors)	Model 1 (SAT)	Model 2 (Institutional factors)	Model 3 (SAT + institutional factors)
**Coefficient for SAT score**	29.207 ***	n/a	19.093 ***	75.686 ***	n/a	40.299 ***
	(3.853)		(6.442)	(5.702)		(10.983)
**N**	264	264	264	264	264	264
***R* ^2^**	0.296	0.378	0.416	00.412	0.525	0.560

Robust clustered standard errors at state level are in parentheses. *** *p* < 0.001, * *p* < 0.05.

**Table 3:** OLS coefficients for SAT predicting college and university rankings.

**Panel A: College Scorecard Data 2016–2017**		**Coefficient for SAT Score**	**Standard Error**	**N**	***R* ^2^**
*THE* U.S. ranking	Model 1 (SAT)	−1.527 ***	0.086	280	0.526
	Model 2 (institutional factors)	n/a		280	0.754
	Model 3 (SAT + institutional factors)	−0.585 **	0.147	280	0.770
Lumosity ranking	Model 1 (only SAT)	−0.847 ***	0.041	204	0.514
	Model 2 (institutional factors control)	n/a		204	0.585
	Model 3 (SAT + institutional factors)	−0.460 ***	0.129	204	0.615
**Panel B: *U.S. News* Data 2014**		**Coefficient for SAT Score**	**Standard Error**	**N**	***R* ^2^**
*THE* U.S. ranking	Model 1 (SAT)	−1.717 ***	0.075	269	0.562
	Model 2 (institutional factors)	n/a		269	0.746
	Model 3 (SAT + institutional factors)	−0.745 ***	0.126	269	0.778
Lumosity ranking	Model 1 (SAT)	−0.929 ***	0.050	199	0.564
	Model 2 (institutional factors)	n/a		199	0.518
	Model 3 (SAT + institutional factors)	−0.742 ***	0.088	199	0.626

Robust clustered standard errors at state level are in parentheses. *** *p* < 0.001, ** *p* < 0.01.

**Table 4:** Proportion of explained variance using Model 1, Model 2, and Model 3.

Variable	Model 1 *R*^2^	Model 2 *R*^2^	Model 3 *R*^2^	$\frac{R_{model\;1}^{2}}{R_{model3}^{2}}$	$\frac{R_{model2}^{2}}{R_{model3}^{2}}$
	**Panel A:** College Scorecard dataset 2017–2018				
6-year salary	0.419	0.569	0.581	0.721	0.979
10-year salary	0.470	0.641	0.653	0.720	0.982
THE U.S. ranking	0.526	0.754	0.770	0.683	0.979
Lumosity ranking	0.514	0.585	0.615	0.836	0.951
	**Panel B:** U.S. News Data 2014				
Early-career salary	0.296	0.378	0.416	0.712	0.909
Mid-career salary	0.412	0.525	0.560	0.736	0.938
THE U.S. ranking	0.562	0.746	0.778	0.722	0.959
Lumosity ranking	0.564	0.518	0.626	0.901	0.827

**Table 5:** OLS Coefficients and importance of predictors of student long-term outcomes and school rankings using Model 3, College Scorecard data.

	Coefficients	Standard Error	Standardized Dominance Statistic	Ranking
	**Panel A:** mean salary six years after graduation			
Average SAT score	16.387 **	5.249	0.188	**2**
Cost per academic year	−0.135	0.096	0.028	8
Admission rate	−3136.935	1883.976	0.016	9
Retention at 4-year-institution	18,836.437 **	6150.669	0.157	**3**
Completion rate	−6873.158	4395.932	0.127	5
Total enrollment	−0.1372 **	0.043	0.040	7
Faculty average salary/month	1.113 ***	0.228	0.152	4
% Students ever borrowed	11325.510	8586.535	0.005	11
% Students received Pell grants	−20,357.645 ***	3811.716	0.221	**1**
Diversity index	82.190 ***	23.575	0.055	6
Location	n/a	n/a	0.010	10
School control	n/a	n/a	0.000	12
	**Panel B:** Mean salary ten years after graduation			
Average SAT score	22.039 **	6.747	0.177	**2**
Cost per academic year	0.005	0.124	0.043	8
Admission rate	−5679.633 *	2421.490	0.026	9
Retention at 4-year-institution	28,917.060 ***	7905.503	0.178	**1**
Completion rate	−2061.146	5650.127	0.149	5
Total enrollment	−0.228 ***	0.055	0.044	7
Faculty average salary/month	1.514 ***	0.293	0.155	4
% Students ever borrowed	10,353.670	11,036.340	0.005	11
% Students received Pell grants	−20,776.170 ***	4899.228	0.163	**3**
Diversity index	103.223 ***	30.302	0.049	6
Location	n/a	n/a	0.011	10
School control	n/a	n/a	0.000	12
	**Panel C:** THE U.S. ranking			
Average SAT score	−0.585 ***	0.130	0.152	4
Cost per academic year	−0.009 ***	0.003	0.119	5
Admission rate	59.200	60.210	0.031	9
Retention at 4-year-institution	−108.600	208.100	0.153	**2**
Completion rate	−543.9 ***	116.100	0.177	**1**
Total enrollment	−0.002	0.001	0.091	6
Faculty average salary/month	−0.033 ***	0.007	0.153	**3**
% Students ever borrowed	203.800	230.400	0.007	11
% Students received Pell grants	−331.400 ***	101.200	0.062	7
Diversity index	−1.249 **	0.569	0.038	8
Location	n/a	n/a	0.019	10
School control	n/a	n/a	0.000	12
	**Panel D**: Lumosity ranking			
Average SAT score	−0.460 ***	0.120	0.258	**1**
Cost per academic year	0.004	0.002	0.046	6
Admission rate	−83.449	51.189	0.023	8
Retention at 4-year-institution	−165.522	202.047	0.171	**3**
Completion rate	−162.704	100.635	0.157	4
Total enrollment	0.001	0.001	0.032	7
Faculty average salary/month	−0.006	0.005	0.068	5
% Students ever borrowed	14.267	183.907	0.006	11
% Students received Pell grants	346.395 **	116.398	0.221	**2**
Diversity index	−0.327	0.646	0.008	10
Location	n/a	n/a	0.009	9
School control	n/a	n/a	0.000	12

Robust standard errors clustered at state level. *** *p* < 0.001, ** *p* < 0.01, * *p* < 0.05.

**Table 6:** OLS coefficients and importance of predictors of student long-term outcomes and school rankings using Model 3, U.S. News data.

	Coefficients	Standard Error	Standardized Dominance Statistic	Ranking
	**Panel A**: Pay-Scale early-career			
Average SAT score	19.092 **	7.011	0.283	**1**
Total enrollment	−0.021	0.038	0.095	5
Acceptance rate	4311.067	2195.387	0.028	8
Average freshmen retention	10,510.41 *	5154.387	0.200	**2**
Endowment	8.82 × 10^−7^ ***	2.25 × 10^−7^	0.119	**3**
Room and board	0.153	0.141	0.044	7
% graduating students ever borrowed loan	−4187.357	3344.803	0.094	6
Average student debt	0.177 *	0.075	0.028	9
Diversity index	89.793 ***	25.093	0.098	4
Location	n/a	n/a	0.008	11
School control	n/a	n/a	0.004	10
	**Panel B**: Pay-scale mid-career			
Average SAT score	40.299 ***	10.757	0.273	**1**
Total enrollment	−0.037	0.074	0.084	4
Acceptance rate	7188.705	4375.667	0.036	8
Average freshmen retention	38,653.78 ***	11,396.04	0.268	**2**
Endowment	9.93 × 10^−7^	6.12 × 10^−7^	0.069	7
Room and board	0.841 **	0.293	0.099	**3**
% graduating students ever borrowed loan	−6762.375	5265.526	0.076	5
Average student debt	0.279 *	0.133	0.015	9
Diversity index	175.039 ***	48.531	0.074	6
Location	n/a	n/a	0.003	11
School control	n/a	n/a	0.005	10
	**Panel C**: THE U.S. Ranking			
Average SAT score	−0.745 ***	0.124	0.253	**2**
Total enrollment	−0.002	0.001	0.120	**3**
Acceptance rate	−72.737	61.716	0.041	8
Average freshmen retention	−1054.54 ***	137.275	0.299	**1**
Endowment	−2.58 × 10^−8^ *	1.09 × 10^−8^	0.084	4
Room and board	−0.014 ***	0.004	0.070	5
% graduating students ever borrowed loan	2.295	71.240	0.043	7
Average student debt	−0.008 ***	0.002	0.020	9
Diversity index	−3.025 ***	0.628	0.053	6
Location	n/a	n/a	0.006	11
School control	n/a	n/a	0.010	10
	**Panel D:** Lumosity ranking			
Average SAT score	−0.742 ***	0.104	0.432	**1**
Total enrollment	−0.001	0.001	0.073	4
Acceptance rate	−142.921 **	53.496	0.042	6
Average freshmen retention	−273.882 *	131.068	0.255	**2**
Endowment	−8.90 × 10^−9^	4.55 × 10^−9^	0.078	**3**
Room and board	−0.004	0.004	0.028	7
% graduating students ever borrowed loan	−27.626	59.679	0.060	5
Average student debt	0.003	0.002	0.018	8
Diversity index	0.616	0.594	0.013	9
Location	n/a	n/a	0.001	10
School control	n/a	n/a	0.000	11

Robust standard errors clustered at state level. *** *p* < 0.001, ** *p* < 0.01, * *p* < 0.05.

## Data Availability

The data was largely drawn from publicly available sources.

## Appendix A

**Table A1:** Summary statistics from college scorecard data 2017–2018.

Variable	N	Mean	SD	Min	Max
SAT average	1300	1060.170	136.849	712	1555
Admission rate	1996	0.672	0.205	0.042	1
Total undergraduate enrollment	6340	2450.591	5509.977	1	100,011
Faculty salary/month	4190	6495.023	2426.603	216	27,570
Retention at 4-year institution	2109	0.729	0.158	0.062	1
% Students ever borrowed loan	5382	0.775	0.222	0.010	0.986
% Students received Pell grant	324	0496	0.222	0	1
Cost per academic year	3590	25,851.390	14,439.030	4259	85,308
Diversity index	707	49.335	17.033	0	89.2
Location	6627	1.814	0.947	1	4
1 = city	3180				
2 = Suburb	2025				
3 = Town	897				
4 = Rural	525				
Control	7068	2.139	0.838	1	3
1 = public	2056				
2 = private non-profit	1970				
3 = private for-profit	3042				
Salary 6 years after graduation	5450	31,383.650	11,626.790	11,800	151,500
Salary10 years after graduation	5259	39,399.140	17,901.610	14,600	250,000
THE U.S. ranking	983	470.236	250.563	1	800
Lumosity ranking	443	226.072	131.155	1	456

**Table A2:** Summary statistics from U.S. News data.

Variable	N	Mean	SD	Min	Max
SAT average	1301	1056.328	121.778	660	1395
Fall 2013 acceptance rate	1293	0.655	0.171	0.077	1
Total enrollment	1200	7856.723	9908.471	161	77,329
Endowment	1073	2.01 × 10^8^	5.64 × 10^8^	65,712	8.27 × 10^9^
Average freshman retention rate	1290	0.751	0.108	0.420	0.970
Room and board	1210	9822.236	2383.622	1032	23,386
% Graduating students ever borrowed loan	962	0.691	0.149	0.083	0.990
Average student debt	947	28,119.670	6373.642	5500	50,275
Diversity index	462	49.138	16.494	9.3	89.2
Location	1106	2.522	1.000	1	4
1 = city	219				
2 = Suburb	287				
3 = Town	404				
4 = Rural	196				
Control	1226	1.782	0.975	1	3
1 = public	478				
2 = private non-profit	745				
3 = private for-profit	3				
Early-career salary	851	44,622.210	5735.902	30,800	68,600
Mid-career salary	851	76,564.390	13,439.65	41,000	131,800
THE U.S. ranking	975	484.673	239.351	15	800
Lumosity ranking	422	238.768	125.014	15	456

**Table A3:** SAT predicting college and university rankings using Model 1 with unrestricted samples.

	Panel A: College Scorecard Data 2016–2017			
	**Coefficient for SAT Score**	**Robust Standard Error**	**N**	***R* ^2^**
U.S. News national ranking	−0.384 ***	0.011	187	0.798
U.S. News Liberal Arts ranking	−0.367 ***	0.014	122	0.825
Revealed preferences ranking	−0.484 *	0.203	92	0.156
THE World ranking	−0.981 ***	0.065	149	0.450
Critical thinking ranking	0.592 ***	0.043	42	0.684
	**Panel B: U.S. News Data 2014**			
	**Coefficient for SAT Score**	**Robust Standard Error**	**N**	***R* ^2^**
U.S. News national ranking	−0.453 ***	0.016	180	0.739
U.S. News Liberal Arts ranking	−0.398 ***	0.020	166	0.741
Revealed preferences ranking	−0.187 ***	0.025	76	0.268
THE World ranking	−0.996 ***	0.126	136	0.284
Critical thinking ranking	0.610 ***	0.034	68	0.716

*** *p* < 0.001, * *p* < 0.05.
